# Mpox outbreak among men who have sex with men in Amsterdam and Rotterdam, the Netherlands: no evidence for undetected transmission prior to May 2022, a retrospective study

**DOI:** 10.2807/1560-7917.ES.2023.28.17.2200869

**Published:** 2023-04-27

**Authors:** Henry J de Vries, Hannelore M Götz, Sylvia Bruisten, Annemiek A van der Eijk, Maria Prins, Bas B Oude Munnink, Matthijs RA Welkers, Marcel Jonges, Richard Molenkamp, Brenda M Westerhuis, Leonard Schuele, Arjen Stam, Marjan Boter, Elske Hoornenborg, Daphne Mulders, Mariken van den Lubben, Marion Koopmans

**Affiliations:** 1 Department of Infectious Diseases, Public Health Service Amsterdam, Amsterdam, the Netherlands; 2 Department of Dermatology, Amsterdam UMC, location University of Amsterdam, Meibergdreef 9, Amsterdam, the Netherlands; 3 Amsterdam Institute for Infection and Immunology, Infectious Diseases, Amsterdam, the Netherlands; 4 Amsterdam Institute for Global Health and Development, Amsterdam, the Netherlands; 5 Department of Public Health, Municipal Public Health Service Rotterdam-Rijnmond, Rotterdam, the Netherlands; 6 Department of Public Health, Erasmus MC University Medical Center, Rotterdam, the Netherlands; 7 Department of Viroscience, Erasmus MC University Medical Center, Rotterdam, the Netherlands; 8 Amsterdam UMC location University of Amsterdam, Department of Infectious Diseases, Meibergdreef 9, Amsterdam, the Netherlands; 9 Amsterdam UMC location AMC, University of Amsterdam, Department of Medical Microbiology and Infection Prevention, Meibergdreef 9, Amsterdam, the Netherlands

**Keywords:** mpox virus, homosexuality, male, epidemics, sexually transmitted diseases

## Abstract

Since May 2022, over 21,000 mpox cases have been reported from 29 EU/EEA countries, predominantly among men who have sex with men (MSM). The Netherlands was the fourth most affected country in Europe, with more than 1,200 cases and a crude notification rate of 70.7 per million population. The first national case was reported on 10 May, yet potential prior transmission remains unknown. Insight into prolonged undetected transmission can help to understand the current outbreak dynamics and aid future public health interventions. We performed a retrospective study and phylogenetic analysis to elucidate whether undetected transmission of human mpox virus (hMPXV) occurred before the first reported cases in Amsterdam and Rotterdam. In 401 anorectal and ulcer samples from visitors to centres for sexual health in Amsterdam or Rotterdam dating back to 14 February 2022, we identified two new cases, the earliest from 6 May. This coincides with the first cases reported in the United Kingdom, Spain and Portugal. We found no evidence of widespread hMPXV transmission in Dutch sexual networks of MSM before May 2022. Likely, the mpox outbreak expanded across Europe within a short period in the spring of 2022 through an international highly intertwined network of sexually active MSM.

**Key public health message**

**What did you want to address in this study?**

The first mpox cases in Europe were reported in the beginning of May 2022, mainly among men who have sex with men (MSM). Yet, it is unknown if we have missed cases before May. We performed a study using stored samples from MSM who visited one of the centres for sexual health in Amsterdam or Rotterdam, the Netherlands, to determine if we had missed cases positive for mpox.

**What have we learnt from this study?**

In 401 anal samples and samples from skin ulcers from visitors who met the current criteria of suspected mpox, we found two positive cases – one in a visitor with an anorectal infection and a second in a visitor with genital ulcers, both sampled in early May 2022 before the first known confirmed mpox case in the Netherlands. The remaining 399 samples, dating as far back as February 2022, were all negative for mpox.

**What are the implications of your findings for public health?**

Our findings suggest that mpox transmission did not occur in Dutch sexual networks of MSM before May 2022, which reflects reports from other European countries including the United Kingdom, Spain and Portugal. The mpox outbreak appeared to spread across Europe over only a short time in the spring of 2022 through an international highly intertwined network of sexually active MSM, soon after COVID-19-related lockdown and travel restrictions were lifted.

## Background

Human monkeypox virus (hMPXV) is an orthopox virus that is closely related to the smallpox virus [1]. It is endemic in Central and West African countries where it has caused recurring outbreaks among humans that have been linked to zoonotic transmission. The reservoirs of hMPXV on the African continent are mainly rodents. In Africa, airborne and close skin-to-skin contact are considered the modes of transmission and cases develop the disease mpox, which is characterised by malaise, airway complaints and a generalised monomorphic rash consisting of vesicles, pustules, and ulcers [2]. Introductions of mpox cases have previously occurred outside known enzootic African countries, but these events have not led to subsequent sustained transmissions [3].

Since May 2022, after initial detection in the United Kingdom (UK), a global mpox outbreak evolved, primarily among men who have sex with men (MSM). The transmission has mainly occurred among MSM with frequent sexual and direct skin contact with multiple partners. Genome sequencing showed that viruses from Clade IIb caused most cases [4].

### Outbreak detection

The first mpox cases of the outbreak in the Netherlands were identified retrospectively on 20 May 2022 in two centres for sexual health (CSH), based on samples from suspected mpox cases seen on 10 May 2022 in Amsterdam, and 19 May in Rotterdam. Since the start of the mpox outbreak, and up to 14 February 2023, 21,178 confirmed cases of mpox have been reported from 29 European Union/European Economic Area (EU/EEA) countries [5]. With 1,260 confirmed cases, and a crude notification rate of 70.7 per million population, the Netherlands was the fourth among countries reporting the most mpox patients since the start of the outbreak. Of all cases, more than 50% occurred in Amsterdam and ca 7% in Rotterdam, both of which have large MSM communities. Moreover, Amsterdam is a popular travel destination for MSM tourists. The weekly number of mpox cases reported in the EU/EEA peaked in July 2022, and since then a steady declining trend has been observed, reaching a plateau with very low numbers since week 52 2022.

The exact moment of introduction and subsequent spread of sexually transmitted mpox among MSM is unknown. Insight into prolonged undetected transmission can help to understand the current mpox outbreak dynamics and aid future public health interventions. Here, we tested a convenience collection of stored anorectal and ulcer samples from MSM visiting the CSH from Amsterdam and Rotterdam on hMPXV, collected between 14 February and 18 May 2022, to determine hMPXV DNA positivity. 

## Methods

### Study design and samples

We analysed convenience samples for hMPXV that had been collected from self-identifying MSM at two CSH, in Amsterdam and in Rotterdam. Since mpox is strongly associated with anorectal hMPXV positivity, and anogenital ulcerative diseases such as genital herpes and syphilis resemble mpox [6], we used anorectal and ulcer samples stored from February to May 2022. Moreover, anorectal hMPXV positivity in asymptomatic individuals has been described [7]. We therefore also included asymptomatic MSM with an anorectal *Neisseria gonorrhoeae* (Ng) and/or *Chlamydia trachomatis* (Ct) infection. As cut-off point, we used in retrospect the day before the first presentation of an identified case in each city, 9 May and 18 May in Amsterdam and Rotterdam, respectively. 

The annual number of consultations in 2018 was 51,128 at the Amsterdam CSH, and 17,445 at the Rotterdam CSH.

### Retrospective analysis in Amsterdam

Anorectal samples are routinely collected from MSM for Ng and Ct testing. All positive samples are stored for 6 months for future reference. Dry samples are collected from visitors with anal, genital or oral (muco)cutaneous ulcers, and tested for herpes simplex virus 1 and 2 (HSV), varicella zoster virus (VZV) and *Treponema pallidum* (Tp). All ulcer swab eluates are stored for 4 months. 

We used qPCR [8,9] to test the Ng- or Ct-positive anorectal samples and all ulcer samples, collected from 14 February to 9 May for the presence of hMPXV. Patient data (i.e. age, symptoms, notified for sexually transmitted infection (STI), STI diagnosed at consultation, pre-exposure prophylaxis (PrEP) use, number of partners in the last 6 months, anal condom use and previous documented STI diagnoses) from the electronic patient file were de-identified before the hMPXV test results became available.

### Retrospective analysis in Rotterdam

Routinely collected anorectal samples from MSM positive for Ng or Ct and ulcer samples tested for HSV1, HSV2 and VZV in the period 1 April to 18 May were tested for the presence of hMPXV. Samples from before 1 April were no longer available. For this purpose, a qPCR assay based either on a pan-orthopox PCR with subsequent hMPXV detection through sequence analysis, or a mpox-specific target was used [8-10]. Patient data (i.e. age, symptoms, notified for STI, STI diagnosed at consultation, PrEP use, partners in the last 6 months, anal condom use, group sex, recreational drug use and previous documented STI diagnoses) were obtained from the electronic patient file.

### Phylogenetic analysis

Whole genome sequencing was performed as described earlier [6] for hMPXV-positive samples. A detailed description of the bioinformatic and phylogenetic analysis can be found in the Supplementary Material S1. For phylogenetic analysis, all available GenBank sequences of hMPXV were downloaded from mPOXSPECTRUM (https://mpox.genspectrum.org). Subsampling was performed in the Nextstrain monkeypox pipeline (https://github.com/nextstrain/monkeypox) with the augur filter settings ’max_date = 2022-06-07’, sequences_per_group = ’1000’ and ‘–exclude-where outbreak!=hMPXV-1’. After filtering, we added the strains from the Netherlands generated as part of this study.

## Results

**Figure 1:**
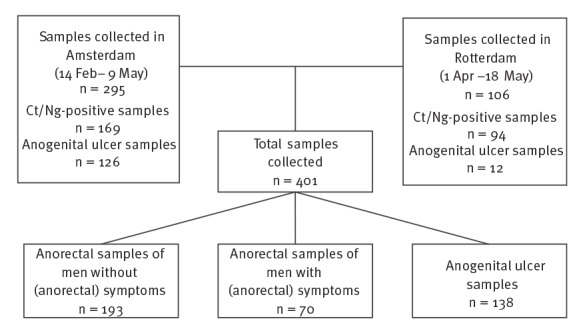
Flowchart of retrospectively collected samples from men who had sex with men visiting the centres for sexual health, Amsterdam and Rotterdam, the Netherlands, February–May 2022 (n = 401)

In Amsterdam, we tested 169 anorectal samples that were positive for Ct/Ng between 14 February and 9 May for the presence of hMPXV (Figure 1, Table 1). At least 40 samples were from visitors with anorectal symptoms. All 169 anorectal samples tested negative for hMPXV. We also tested all 126 ulcer samples (irrespective of test result for HSV, VZV or Tp) collected from 14 February to 9 May. In the ulcer selection, we identified one hMPXV-positive sample collected on 6 May 2022. 

Ct: *Chlamydia trachomatis*; Ng: *Neisseria gonorrhoeae*.

**Table 1:** Monkeypox virus test results of samples from men who had sex with men visiting centres for sexual health, Amsterdam and Rotterdam, the Netherlands, February–May 2022 (n = 401)

Sample type	Location	Test results		
		Negative	Positive	Total
Anorectal samples of men without anorectal symptoms^a^	Amsterdam	129	0	129
	Rotterdam	64	0	64
Anorectal samples of men with anorectal symptoms^a^	Amsterdam	40	0	40
	Rotterdam	29	1	30
Positive anogenital ulcer samples^b^	Amsterdam	79	0	79
	Rotterdam	4	0	4
Negative anogenital ulcer samples^c^	Amsterdam	46	1	47
	Rotterdam	8	0	8
Total		399	2	401

^a^ Rotterdam: any symptom, Amsterdam: anorectal symptoms (proctitis and/or skin lesions).

^b^ Ulcer samples positive for either herpes simplex virus (Amsterdam and Rotterdam), or *Treponema pallidum* or *Chlamydia trachomatis* (Amsterdam).

^c^ Ulcer samples negative for either herpes simplex virus (Amsterdam and Rotterdam), or *Treponema pallidum* and *Chlamydia trachomatis* (Amsterdam).

In Rotterdam, we tested 94 anorectal samples for hMPXV from visitors positive for Ct/Ng, of whom 30 reported symptoms in the period 1 April to 18 May (Figure 1, Table 1). We found one hMPXV-positive anorectal sample collected on 9 May 2022 from an MSM who reported symptoms of proctitis. In the same period, an additional 12 ulcer samples were also tested for hMPXV; all tested negative.

The patient characteristics are presented in Table 2. A man in his early 50s with multiple ulcers and an itchy rash on his upper legs and a man his early 20s with proctitis both tested positive for hMPXV.

**Table 2:** Disease and sexual behaviour characteristics of two mpox-positive men who had sex with men visiting centres for sexual health, Amsterdam and Rotterdam, the Netherlands, May 2022

Date of visit	Location	Age (years)	Symptoms	Notified for STI	STI diagnosed at consultation	PrEP use	Partners in the last 6 months (n)	Anal condom use	Group sex	Recreational drug use	Previous documented STI diagnoses
6 May 2022	Amster-dam	50s	Multiple ulcers and itchy rash on upper legs	No	Genital herpes	NA	1	Sometimes	Unknown	Unknown	HIV, Ct, Ng and primary syphilis
9 May 2022	Rotter-dam	20s	Proctitis	Yes	Anorectal and pharyngeal Ct and Ng	Yes	30	Sometimes	Yes	Yes	Yes

Ct: *Chlamydia trachomatis*; NA: not applicable; Ng: *Neisseria gonorrhoeae*; PrEP: pre-exposure prophylaxis against HIV; STI: sexually transmitted infections.

### Phylogenetic analysis

**Figure 2:**
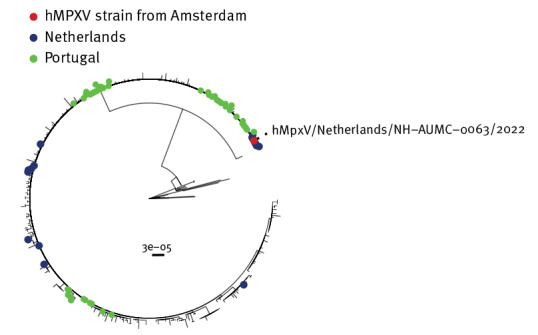
Phylogenetic association of one human monkeypox virus strain in a sample from a visitor at the centre for sexual health, Amsterdam, the Netherlands, 6 May 2022 and reference strains from the Netherlands (n = 11) and Portugal (n = 48) up to 7 June 2022

The two hMPXV-positive samples were retrieved from the storage for phylogenetic analysis. Unfortunately, one of the hMPXV samples (collected in Rotterdam) contained too little DNA to perform successful sequence analysis. The positive sample from Amsterdam (6 May 2022) was sequenced and the identified strain was compared to all available hMPXV sequences from the Netherlands (Figure 2). Results indicated that the identified strain belonged to the Clade IIb cluster (B.1) and was closely related to the strains from the Netherlands, as well as to strains of hMPXV from Portugal (Figure 2).

hMPXV: human monkeypox virus.

Additional reference hMPXV sequences from the Netherlands and Portugal were obtained via https://mpox.genspectrum.org**,** and combined with the obtained sequence from this study. For more detailed information, please see Supplementary Material S1.

## Discussion

We identified two symptomatic mpox cases on 6 May and 9 May 2022 that preceded the first identified cases (10 May 2022) in the Netherlands. We did not detect hMPXV in the other 399 of 401 samples predating the first cases. Our study included samples from 208 MSM with complaints suspected of mpox according to the current WHO suspected case definition criteria [11], such as anogenital ulcers (n = 138) and proctitis (n = 70). We therefore assume it unlikely that substantial undetected transmission of hMPXV occurred in the Netherlands before 6 May 2022. The mean incubation period for cases identified in the Netherlands was 8.5 days (95% credible intervals (CrI): 6.6–10.9) [12]. These findings suggest that the introduction of hMPXV in Dutch sexual networks of MSM started sometime at the end of April 2022. This coincides with the earliest symptom onset of mpox cases in the UK on 21 April [13,14], in Spain on 26 April [15] and in Portugal on 29 April [16]. In combination with the phylogenetic analysis of hMPXV genome sequences, which showed that the initial cases across Europe are clonal, it is likely that the mpox outbreak expanded internationally within a short period (weeks) in the spring of 2022 in an international highly intertwined network of sexually active MSM. The mpox outbreak also coincided with international relaxation of COVID-19 prevention measures and resumption of global travel [17]. 

Both cases had characteristics that are associated with increased risk of spread of STI such as multiple previous STI, multiple sex partners, condomless anal sex, either HIV PrEP use or living with HIV and recreational drug use. These characteristics predispose individuals to STI and to mpox [3,6]. 

The strength of our study was that we had an extensive number of samples from anogenital lesions, and anorectal samples available from persons who met the current case definition for mpox. In addition, we included asymptomatic patients, which was of interest since asymptomatic hMPXV carriership has been described during the 2022 outbreak [7]. However, a limitation was that the group tested was biased, as the anorectal samples were only stored if they tested positive for Ct and/or Ng. Also, because of privacy regulations, the study was anonymous. Hence, we could not gather additional data on date of symptom onset, recent sexual partners and travel history. In this mpox outbreak in MSM, it remains unclear how hMPXV was introduced. Apart from network effects [18], the strains circulating in 2022 differ from previous hMPXV strains by over 50 nucleotides. Whether such changes led to increased sexual transmission is not yet known. Further viral characterisation is needed as well as continued genomic surveillance on circulating hMPXV strains [4].

## Conclusions

We found no indication of extensive undetected transmission of mpox among MSM in Amsterdam or Rotterdam before May 2022. This is in accordance with findings from other European cities with large MSM populations, and is in support of a clonal international mpox outbreak in the spring of 2022.

## Supplementary Data

113000 Supplement
