# Supplementary material for: Mpox outbreak among men who have sex with men in Amsterdam and Rotterdam, the Netherlands: no evidence for undetected transmission prior to May 2022, a retrospective study
Source: Euro Surveill. 2023 Apr 27;28(17):2200869. doi: 10.2807/1560-7917.ES.2023.28.17.2200869 (PMC10283470; doi:10.2807/1560-7917.ES.2023.28.17.2200869)
Supplement: Supplement [file 22-00869_deVRIES_SUPPLEMENT.pdf]

Supplementary material S1

This supplementary material is hosted by Eurosurveillance as supporting information alongside the article Mpox outbreak among men who have sex with men in Amsterdam and Rotterdam, the Netherlands: no evidence for undetected transmission prior to May 2022, a retrospective study on behalf of the authors who remain responsible for the accuracy and appropriateness of the content. The same standards for ethics, copyright, attributions and permissions as for the article apply. Eurosurveillance is not responsible for the maintenance of any links or email addresses provided therein.

Bioinformatic analysis

Short (<100bp) and very long reads (>4000bp) were initially removed from the raw fastq passed reads. All remaining reads were subsequently mapped to the human genome hg19 to remove contaminant human reads using minimap2 version 2.18-r1015 with default settings[1]. Unmapped reads were remapped against reference sequence MPXV_USA_2022_MA001 (Genbank accession number ON563414.2). The resulting sam-file was coverted to bam-file format, sorted and indexed using samtools v.1.10[2]. A consensus sequence was generated using the TrueConsense package (https://github.com/RIVM-bioinformatics/Trueconsense) using settings (-cov 30 -noambig). In case of single gaps/deletions a detailed coverage file was generated per position. For said positions the deletion was replaced by the most dominant remaining A/C/T/G-nucleotide in case both forward and reverse orientation matched and both orientations had a minimal coverage of 30. If one of these conditions was not met, the deletion nucleotide would be replaced by an "N". Finally, all homopolymer regions were manually checked for inconsistencies. The resulting consensus sequences of the described cases was deposited in GISAID.

Phylogenetic analysis

Additional hMpxV sequences were obtained via mpox-spectrum.org (https://mpox.genspectrum.org/) by downloading the available pre-aligned sequences (representing all available sequences obtained worldwide) downsizing to sampling dates prior to June 7th 2022 and that dataset was combined with the obtained sequences from this study. Phylogenetic analysis was then performed using the nextstrain[3] monkeypox build (https://github.com/nextstrain/monkeypox). Phylogenetic tree was rooted to the sequence of Genbank accession number MK783032 and visualized using the ggtree2 package in R version 4.0.3[4,5]. Final editing of the tree was done manually using Adobe Illustrator CS6.

References

1. Li H. Minimap2: Pairwise alignment for nucleotide sequences. Bioinformatics 2018;34(18):3094–100.
2. Li H, Handsaker B, Wysoker A, et al. The Sequence Alignment/Map format and SAMtools. Bioinformatics 2009;25(16):2078–9.
3. Hadfield J, Megill C, Bell SM, et al. NextStrain: Real-time tracking of pathogen evolution. Bioinformatics 2018;34(23):4121–3.
4. R Development Core Team. R: A language and environment for statistical computing [Internet]. 2008;Available from: http://www.r-project.org
5. Yu G, Smith DK, Zhu H, Guan Y, Lam TTY. Ggtree: an R Package for Visualization and Annotation of

Phylogenetic Trees With Their Covariates and Other Associated Data. Methods Ecol Evol 2017;8(1):28–36.
